# Retrospective Analysis of Movement Data Before and After an Ankle Fracture: A Descriptive Study Using Apple Health

**DOI:** 10.1016/j.mcpdig.2026.100362

**Published:** 2026-04-24

**Authors:** Erik Börjesson, Emilia Möller Rydberg, Carl Bergdahl, Sebastian Andreasson, Torbjörn Lundh, Michael Möller, David Wennergren

**Affiliations:** a Institute of Clinical Sciences, Sahlgrenska Academy, University of Gothenburg, Gothenburg, Sweden; b Department of Orthopaedics, Sahlgrenska University Hospital, Gothenburg Region Västra Götaland, Sweden; c Centre for Digital Health, Sahlgrenska University Hospital, Gothenburg Region Västra Götaland, Sweden; d Sahlgrenska Academy, Department of Applied IT, University of Gothenburg, Sweden; e Theoretical Sciences Visiting Program, Okinawa Institute of Science and Technology, Okinawa, Japan; f Department of Mathematical Sciences, University of Gothenburg, Gothenburg, Sweden; g Department of Mathematical Sciences, Chalmers University of Technology, Gothenburg, Sweden

## Abstract

**Objective:**

To evaluate movement patterns in patients with ankle fractures before and after injury.

**Patients and Methods:**

This descriptive study analyzed movement data from patients treated for ankle fractures at Sahlgrenska University Hospital, Sweden (January 1 to December 31, 2022). Patients were identified using ICD-10 codes and medical records. Inclusion criteria: surgical and nonsurgically treated patients with >6 months of preinjury iPhone use. Step count, length, and speed were collected through a mobile application integrated with Apple Health. Double support and gait asymmetry were excluded due to limited external validity. Data spanned 6-12 months preinjury to 1 year post-injury. The primary aim was to evaluate whether patients reach their preinjury movement patterns.

**Results:**

Of 1131 patients, 90 were analyzed. Preinjury means: 5435.0 steps (SD 4215.3), step length 0.70 m (SD 0.07), and step speed 1.28 m/s (SD 0.2). At 1 year: 5420.3 steps (SD 3887.0), step length 0.68 m (SD 0.08), and step speed 1.22 m/s (SD 0.19). A post-injury plateau was reached in step parameters at 84.8 days, with no further recovery thereafter. Step count largely recovered, but deficits in step length and speed persisted at 12 months.

**Conclusion:**

Smartphone-derived movement data provide a cost-effective alternative to laboratory gait analysis, enabling long-term monitoring. Preinjury data allow individualized baseline comparisons and may support earlier identification of patients needing adjusted rehabilitation.

Ankle fractures are common across all ages and activity levels.^1^, ^2^, ^3^ They can lead to long-term disability, mobility limitations, and decreased quality of life.^4^^,^^5^ Stable ankle fractures are generally treated nonsurgically with a cast, orthosis, or elastic bandage, while unstable fractures are typically managed surgically with plates and screws.^6^

Most studies assess ankle fracture outcomes using ankle range of motion and radiology; a few studies have focused on patient-reported outcomes.^5^^,^^7^, ^8^, ^9^ Many individuals improve substantially after an ankle fracture, but newer studies suggest that movement or functional patterns may not fully reach preinjury levels, especially in surgically treated patients.^10^^,^^11^ To date, there is a lack of research focusing on comprehensive objective data, beginning from the preinjury phase and continuing throughout the first year of recovery after an ankle fracture.

Today, most of the global population owns a smartphone or a smartwatch.^12^ These devices feature a built-in step counter and accelerometers that continuously collect information about users’ daily activity and movement patterns. Data from these devices can be collected and analyzed, providing insights into the user’s everyday activity and movement patterns. The Apple Health application, introduced with iOS 8, is preinstalled on all iPhones and automatically collects data on various movement parameters, including step count, step length, speed, distance, and the number of floors climbed, storing the information continuously. The validity and accuracy of data from the Apple Health application have been confirmed in several studies.^13^^,^^14^

Restoring walking ability is regarded by many patients as one of the most important outcomes after an ankle fracture. Derived from smartphone-based devices, these movement parameters reflect overall gait ability and can help identify clinically relevant gait disturbances that might not be detected through traditional patient-reported outcomes or radiographic imaging.

The primary aim of this study was, from an exploratory perspective, to analyze the impact of an ankle fracture on individuals’ normal movement patterns, and the timeline for the restoration of preinjury movement patterns after the injury. Secondary aims were to investigate whether other factors (eg, fracture type, age, or sex assigned at birth) affect the return to preinjury movement patterns.

## Patients And Methods

### Study Setting

This retrospective observational cohort study analyzed movement recovery in adults after an ankle fracture. Participants were recruited from Sahlgrenska University Hospital, Gothenburg, Sweden, which is the only emergency hospital for orthopedic trauma patients in the Gothenburg metropolitan area. Eligible for the study were all patients aged ≥16 years who had been iPhone users for over 6 months before the injury, with a radiologically verified ankle fracture treated at SU from January 1, 2022 to December 31, 2022. A fracture of the ankle involves one or more of the malleoli of the tibia and the fibula and is sometimes accompanied by damage to adjacent ligament structures. All fractures were classified according to the AO classification system (Type A, B, and C), and the orthopedic surgeon determined treatment. Treatment was categorized as nonsurgical or surgical. Exclusion criteria included polytrauma, significant cognitive impairment, non-iPhone use, or inability to speak and understand Swedish.

### Study Design

Data collection took place from February 2024 to December 2024 through medical records, relevant ICD-10 codes, and an iPhone mobile application. To facilitate data collection from patients, a mobile application known as Brytpunkten (The Breaking Point) was created in collaboration with colleagues from Gothenburg University's Human-Computer Interaction department in Sweden for this study. Patients who met the inclusion criteria were invited to download the application and participate in the study. Selection bias was mitigated by contacting all eligible patients who sustained an ankle fracture in 2022. To address measurement bias, only the validated Apple Health application was used to collect movement data. The diverse methodologies employed by Android smartphone manufacturers for collecting movement data introduce a potential for bias. Therefore, confidence in the validity of data from other devices was difficult due to quality concerns.

Data obtained from the mobile application through the Apple Health application encompassed: (1) step count, (2) step length, and (3) step speed. Movement parameters such as double support time, gait asymmetry, walking and running distance were not collected due to concerns about external validity. The data collection period spanned from 6-12 months before the injury to 12 months after. In total, 81 (90%) of the patients had movement data spanning at least 1 year before the injury. The information gathered from the mobile application was pseudonymized and stored on an encrypted server at GU in compliance with general data protection regulations standards and Swedish law. This study was approved by the Swedish Ethical Review Authority (reference number 2024-00068-01).

### The iPhone Mobile Application

On installing the application, the participants shared their Apple Health data, which was uploaded in a JavaScript Object Notation format to a secure server at the University of Gothenburg. The data was processed and analyzed using Python with Jupyter Notebooks. The application was built for iOS to specifically extract data from Apple Health. To address smartphone-carrying bias, days with no data (eg., when the phone was turned off) were excluded. Additionally, for users with multiple devices, such as an iPhone and an Apple Watch, data from the device with the most recorded parameters was used.

### Patient Inclusion Process

A total of 1131 patients were identified using medical records and relevant ICD-10 codes. Each medical record was reviewed to extract patient characteristics, such as treatment, and to confirm that the inclusion criteria were met. Subsequently, 783 patients were invited to voluntarily participate in the study by telephone and letter. Around half of these individuals were iPhone users. The letter included a link to the mobile application and study information, where the Brytpunkten application could be downloaded. Patients were required to sign a digital consent before using the application. All patients received information about the study and gave written consent. Participation was voluntary, and participants could withdraw at any time. Data were managed under general data protection regulations, and all data were anonymized before analysis. Finally, 110 patients enrolled in the study and downloaded the iPhone mobile application. See Figure 1.

### Statistical Methods

Changes in movement parameters were interpreted relative to the standard error of measurement (SEM). As no reliable threshold exists for smartphone-derived movement parameters, SEM values were derived from previously published inertial-sensor-based gait analysis in patients with bimalleolar ankle fractures.

The minimal detectable change at the 95% CI (MDC95), calculated using the formula (MDC=1.96 × √2 × SEM), was used to determine whether changes exceeded measurement error. Changes exceeding the MDC95 were considered to represent true change beyond measurement error. The step count was interpreted descriptively due to a lack of validated SEM or MDC thresholds for iPhone-derived movement parameters in the literature. The SEM values for walking speed and step length were 0⋅02 m/s and 0⋅02 m (MDC95=0.055), respectively.^15^

To estimate the different phases of recovery of patients’ movement, daily step count was modeled as a piecewise-linear (broken-stick) function of time relative to injury. The model defines 3 phases: a preinjury plateau a, a linear recovery phase, and a post-injury plateau b. The recovery duration c defines the transition point between linear increase and post-injury plateau. f(x)= a if x < 0, to (b/ c)·x if 0 ≤ x < c, b if x ≥ c.

The model was fitted individually to each participant using nonlinear least squares. Of the 90 participants, stable answers were received from 87 (95.6%), confirming model convergence. Model parameters were estimated using a bounded nonlinear least square from the Python (version 3.13) package. Initial parameter estimates were derived from the mean preinjury and post-injury step counts, along with a default recovery duration of 30 days. Descriptive statistics were reported as counts (%) for categorical variables and means ± SD for continuous variables. The (broken-stick) statistical model was not used to differentiate phases in step speed or step length, and these variables were handled descriptively.

## Results

Total of 1131 patients with ankle fractures were screened using medical records. Before data analysis, 348 patients were excluded because of incorrect diagnosis (n=134, 38.5%), patient fatality (n=40, 11.5%), fracture in a year other than 2022 (n=86, 24,7%), treatment abroad (n=53, 15.2%), cognitive impairment (n=11, 3.1%), and missing contact information (n=24, 6.9%). The initial eligibility criteria were satisfied by 783 participants. Of these 783 participants, 110 (14.0%) were successfully included, downloaded the application, and provided their movement data. After data analysis, 20 participants were excluded due to insufficient data quality (n=13, 65.0%), and technical difficulties encountered during app setup (n=7, 35.0%). The final analysis included 90 participants.

The mean age at injury was 52 years (SD 16.1). The cohort comprised 64 women (SD 71.1%) and 26 men (28.9%). The mean age at the time of injury was higher in women (55.30 years, SD 14.5) than men (47.15 years SD 18.5). Most patients had a type B ankle fracture (n=57, 63.3%), followed by type A (n=28, 31.1%), and type C fractures (n=5, 5.6%). Surgical treatment was administered to 44 (48.8%) patients and 46 (51.2%) received nonsurgical treatment. (See Table 1 for baseline characteristics of study population.)

**Table 1:** Baseline Characteristics of the Study Population

Characteristics	All fractures N=90 (100%)	Type A n=28	Type B n=57	Type C n=5
% of all cases		31.1%	63.3%	5.6%
Sex assigned at birth, n (%)				
Male	26 (28.9%)	7 (25.0%)	16 (28.1%)	3 (60.0%)
Female	64 (71.1%)	21 (75.0%)	41 (71.9%)	2 (40.0%)
Age, mean (range)				
Total	52.9 (16-79)	49.7 (16-79)	55.0 (19-79)	47.6 (37-62)
Male	47.1 (16-77)	36.1 (16-65)	52.0 (19-77)	46.6 (37-62)
Female	55.3 (20-79)	54.2 (21-79)	56.1 (20-79)	49.0 (39-59)
Age groups in y				
16-39, n (%)	18 (20.0%)	7 (25.0%)	9 (15.7%)	2 (40.0%)
40-59, n (%)	35 (38.8%)	11 (39.3%)	22 (38.6%)	2 (40.0%)
60-99, n (%)	37 (41.2%)	10 (35.7%)	26 (45.6%)	1 (20.0%)
Treatment, n (%)				
Surgical treatment, n (%)	44 (48.8%)	3 (10.8%)	37 (65.0%)	4 (80.0%)
Nonsurgical treatment, n (%)	46 (51.2%)	25 (89.2%)	20 (35.0%)	1 (20.0%)

### Recovery of Movement Parameters

**Figure 2:**
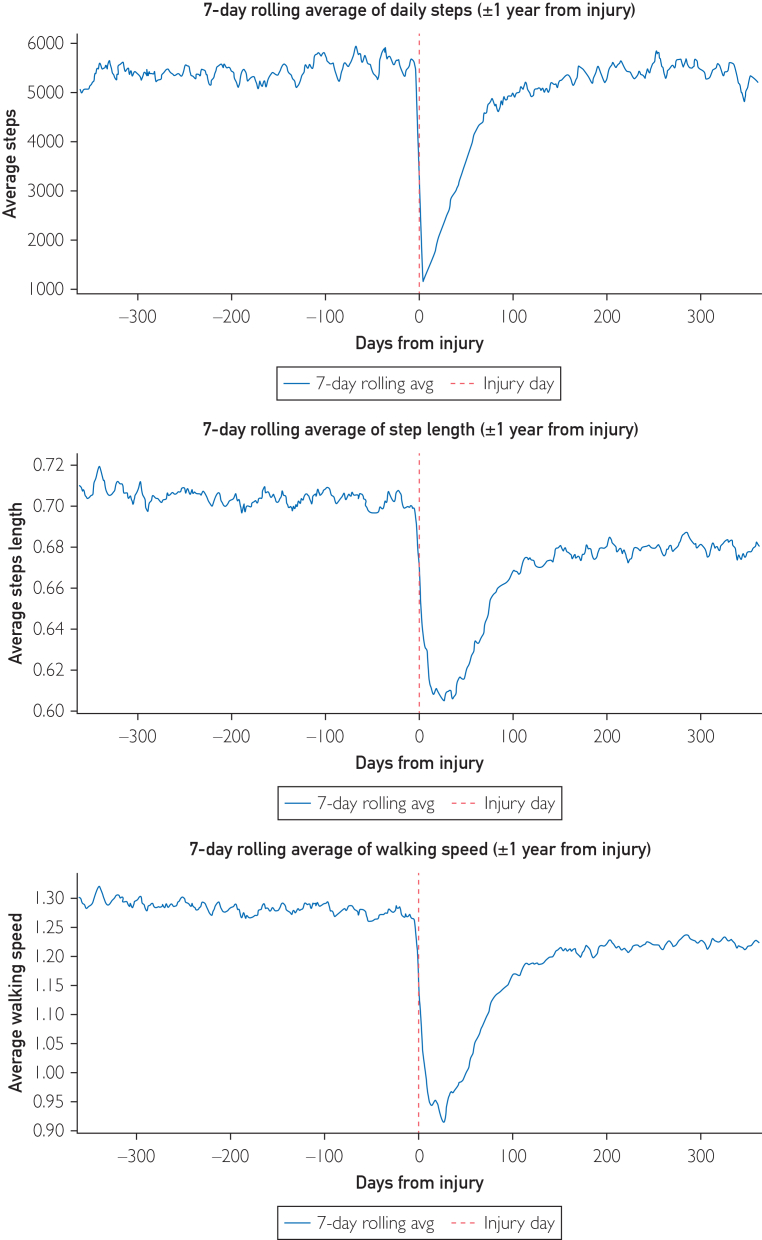
Mean number of steps, step length and step speed taken by all participants (N=90) in the year preceding and following an ankle fracture.

The mean number of steps taken per day before their ankle fracture was 5435.0 (SD 4215.3, 95% CI, 4836-6074). After the fracture, the mean values were 1797.5 steps (SD 2491.1) at 0-30 days, 3373.7 steps (SD 3159.2) at 31-60 days, and 4628.9 steps (SD 3559.6) at 61-90 days (Figure 2). At 1 year post-injury, the mean steps was 5420.3 (95% CI, 4943-6045) per day.

The average step length before the ankle fracture was 0.70 m (SD 0.07). During the first 30 days, the value was 0.62 m (SD 0.11); from 31 to 60 days, it remained at 0.62 m; and from 61 to 90 days, it increased to 0.65 m (SD 0.09). A gradual increase in step length was observed over the course of the observation period (0.62 m at baseline, reaching 0.65 m after the initial 90 days). At 1 year post-injury, the mean step length was 0.68 m. This reduction did not exceed the minimal detectable change at the 95% CL (MDC95=0.055 m). See Figure 2.

The mean walking speed 1 year before the injury was 1.28 m/s (SD 0.2). From 0 to 30 days, the mean walking speed was 0.97 m/s (SD 0.3); from 31 to 60 days, it was 0.99 m/s (SD 0.2); and from 61 to 90 days, it was 1.11 m/s (SD 0.2). The average walking speed from 0 to 90 days after the fracture decreased by 0.17 m/s (from 1.28 to 1.11). At 1 year post-injury, walking speed was 1.22 m/s. This reduction did exceed the minimal detectable change at the 95% CI (MDC95=0.055 m/s). See Figure 2.

**Figure 3:**
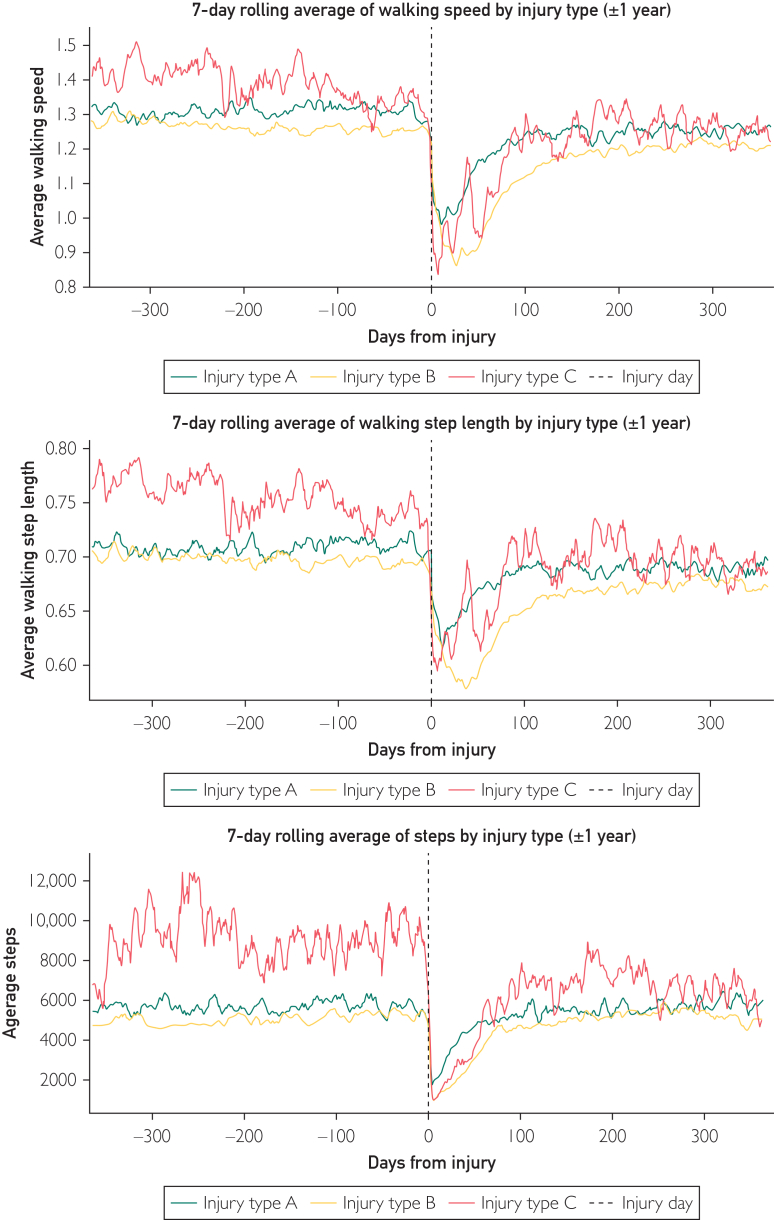
Average step count, walking speed, and length of fracture type A, B and C, 1 year before and after an ankle fracture.

A comparison of fracture types revealed that Type A fractures decreased stride length from 0.71 m to 0.69 m and speed from 1.31 m/s to 1.25 m/s, whereas step count remained relatively unchanged, at 5678 to 5665. Type B fractures reduced stride length from 0.70 m to 0.67 m and walking speed from 1.26 m/s to 1.20 m/s. The step count remained relatively constant, ranging from 5092 to 5286. Type C fractures reduced their step count from 9085 to 7300. See Figure 3.

The post-injury plateau phase in step count recovery was reached after an average of 85 days (95% CI, 72.8-96.9). Type A fractures reached the plateau phase in 47.4 days (95% CI, 33.9-60.9), Type B in 99.9 days (95% CI, 84.8-115), and Type C in 118 days (95% CI, 5.8-230.2). While the average step count of Type A and B fractures remained consistent 1 year after the fracture, Type C fractures exhibited a decline from 9085.0 to 7300.0 steps. Both men and women reached their post-injury plateau phase at ∼85 days. However, younger participants (<65 years) reached their plateau phase faster than those aged ≥65, at 73.7 days (95% CI, 60.3-87.4) and 103.6 days (95% CI, 80.0-125.0), respectively (Table 2).

**Table 2:** Average Number of Steps Pre-Ankle and Post-Ankle Fracture, Categorized by Fracture Type, Age Group, and Sex Assigned at Birth, as well as the Duration to Achieve the Post-Injury Plateau Phase During Recovery

Groups	Time to Reach the Post-Injury Plateau Phase (D)	95% CI	Number of Steps Preinjury	95% CI	Number of Steps Post-Injury	95% CI
All patients	84.8	72.8-96.9	5435	4836-6074	5420	4943-6045
Fracture type						
Type A fracture	47.4	33.9-60.9	5678	4661-6695	5665	4833-6498
Type B fracture	99.9	84.8-115.0	5092	4372-5812	5286	4534-6037
Type C fracture	118	5.8-230.2	9085	294-17,877	7300	5116-9483
Sex assigned at birth						
Male	85.4	60.5-110.4	6415	4924-7907	6750	5508-7993
Female	84.6	70.5-98.7	5039	4425-5653	4950	4402-5497
Age groups						
<65 y	73.7	60.3-87.4	5926	5173-6661	6033	5310-6734
>65 y	103.6	80.0-125.0	4660	3626-5800	4584	3816-5475

## Discussion

This study demonstrates that most patients experience a satisfactory recovery in step count, but not in step length and speed, 12 months after the trauma. This study represents the first to analyze movement patterns in individuals with ankle fractures. The study covers the year preceding the trauma and the first year of recovery, using Apple Health data. A post-injury plateau was reached in step parameters at 84.8 days, with no further recovery thereafter. Notably, neither step speed nor step length showed further progress beyond this point. This implies that even with recovered step counts in A and B fracture types, patients’ recovery is incomplete. Our data may offer valuable insights to patients and health care providers on movement patterns before injury and changes that occur during rehabilitation.

### Strengths

The study’s major strength lies in the acquisition of unbiased movement data spanning the time before the injury through 1 year post-injury. Due to the study’s retrospective design, patients were not informed of future data collection, which provided unique insights into movement and general activity levels years before the injury and throughout the recovery phase. Approximately half of all smartphone users in Sweden, where roughly 95% of those over 16 years old use smartphones, are iPhone users from diverse demographics.^16^

### Limitations

The most important limitation is that obtaining movement data from a smartphone or smartwatch hinges on the user’s possession of the device. Without this device, data cannot be collected. The study excluded 13 individuals whose data did not meet quality standards, predominantly due to participant behavior. This included not carrying their smart devices or disabling their Apple Health application, which precluded the acquisition of movement data. Another limitation is that the study only included iPhone users. Despite representing over 50% of the total smartphone user population, this could result in the omission of data from other smartphone users.^17^ However, iPhone users in Sweden are evenly distributed across age and sex groups and thus do not present any apparent differences when compared with Android users.^16^ Another limitation is the small number of patients in the type C fracture category (n=5).

### Validity of Apple Health

The Apple Health application on the iPhone has been validated in several studies and is considered a reliable tool for evaluating daily steps, walking speed, and step length in everyday activities, particularly in adults and seniors.^13^ Data accuracy is further enhanced when integrated with a smartwatch.^14^^,^^18^

### Step Count

In fracture types A and B, the step count returned to preinjury levels within one year, including more complex fractures that required surgical intervention. Type C fractures, however, showed a reduction in step count, yet a greater number of steps were recorded before the trauma. Stigevall et al^5^ reported that type C fractures are associated with a poorer prognosis and that patients tend to be younger. Before the injury, the study cohort’s activity level was roughly 6000 steps per day, higher than the global average of 5000 steps per day.^19^ Nevertheless, these figures correlate with extant Swedish population data, with a mean daily step count of 6,000 (16). Increasing evidence suggests that step counters may help identify patients at risk of delayed recovery after injuries, even as early as 1 month post-injury. Enhanced patient outcomes can be attained by prioritizing these individuals and providing comprehensive rehabilitation support.^20^

### Step Length

Across all fracture types, the average step length declined from 0.70 m before injury to a post-injury steady state of 0.68 m per step, indicating a 0.02 m reduction in mean step length at one year. Directly after the trauma, step length remained at 0.62 m for the initial 60 days; however, a gradual increase of 0.03 m was observed between days 60 and 90, ultimately reaching a post-injury steady state. The reduction in step length (−0.02 m) did not exceed the MDC threshold (MDC95=0.055 m), suggesting variability rather than a definitive impairment. Step length is regarded as an important component of a person’s overall gait and is reported to contribute to gait speed.^21^ Thus, a shorter step length could result in a lower overall step speed.^21^ Evidence suggests that low step length and speed are linked to an elevated risk of falls among older adults (age ≥65 years).^22^ Espy et al^21^ differentiated between walking speed and walking length, concluding that reduced step length correlated with diminished confidence in ambulation.^21^ This assertion may be valid after an ankle fracture. Hancock et al^8^ found that patients with an ankle fracture who exhibited reduced ankle dorsiflexion after cast removal also showed lower outcome measures at a 6 month follow-up. The authors concluded that this might affect ankle function.^8^ Additionally, Aquino et al^23^ demonstrated that limited ankle dorsiflexion resulted in shorter step lengths during ambulation, potentially contributing to the compromised step length observed in these patients.^23^

### Step Speed

The mean walking speed of participants with all fracture types decreased from 1⋅28 m/s to 1⋅11 m/s between the pre-trauma and 90-day post-injury assessments. The greatest recovery occurred between days 61 and 90, with an increase from 0.99 m/s to a new peak at 1.11 m/s. At 1 year post-injury the step speed was 1.22 m/s. An observed reduction in step speed was seen (0.06 m/s); that exceeded the minimal detectable change (MDC 95=0.055 m/s), suggesting that the observed difference may be clinically meaningful at the individual level. As previously noted, an ankle fracture can adversely affect both step length and speed, as these factors are interdependent. Walking speed analysis is increasingly recognized as a reliable indicator of overall health. A slow walking speed has been associated with a higher risk of falls, hospital admissions, lower limb disability, and overall cause of mortality.^24^ Finally, Dumurgier et al^25^ showed that people with slower gait had a threefold increase in the likelihood of cardiovascular death. Schoenmakers et al^26^ showed that patients with trimalleolar fractures reduced their walking speed and range of motion after the injury, suggesting that more complex fractures have poorer outcomes. Our study patients exhibited a reduction in walking speed that persisted for 12 months after the injury event. Whether this will affect their long-term general health remains to be determined.

### Interpretation

This study shows that while most patients treated for an ankle fracture achieve satisfactory recovery in step count, their step length and step speed remain compromised 12 months after the trauma. Reduced step length may be attributed to decreased range of motion and psychological factors (eg, fear of falling, anxiety) following ankle trauma, thus reducing step speed. Once the patient attains the post-injury plateau, typically around 84.8 days, there is negligible improvement in step movement parameters. This observation suggests that the first 3 months post-injury are critical for rehabilitation. Targeted early rehabilitation for select individuals after trauma may improve patient recovery.

### Generalisability

Our findings may be applicable to adults in comparable populations who have access to smartphones, which represent about 60% of the global population in 2024.^12^ However, generalisability to those with limited smartphone access may be limited. Although this study was conducted at a single center and included only iPhone users, the demographic profile of our participants closely mirrors that of the general Swedish population, as well as findings from previous large demographic studies in Sweden.^1^ We found no apparent skewness between our study population and broader Swedish demographics; Therefore, we believe our results are likely applicable or useful to a broader range of people. Future studies are needed to confirm these findings across different populations and healthcare systems, including those with limited health literacy.^27^

### Implications for Clinical Use

This study’s analysis of Apple Health movement data revealed that patients with ankle fractures did not recover their step length and speed post-trauma. However, for most patients, the step count returned to preinjury levels after the trauma event. As previously mentioned, this finding suggests that the most significant improvement in movement parameters happens within the first three months after the trauma; therefore, it may be beneficial to target certain individuals early in their rehabilitation to facilitate a more efficient recovery. These parameters could serve as additional information when assessing the recovery of a patient suffering from an ankle fracture. Routinely collecting data for each case can help identify patients who may need extensive rehabilitation early, potentially improving recovery outcome. The study format, which examines movement patterns, could prove valuable in evaluating the rehabilitation of a wider array of lower extremity traumas that impact gait and overall mobility in the future.

## Conclusion

This study offers innovative evidence on patient mobility before and during recovery from an ankle fracture. While most patients show satisfactory recovery in step count, reductions in step length and walking speed remain evident even 12 months after trauma. Future studies should aim to validate these findings in larger cohorts. The use of smartphone-derived movement data offers a cost-effective and accessible alternative to laboratory-based gait analysis, enabling long-term follow-up in patients' everyday settings. Access to preinjury movement data also allows recovery to be interpreted relative to each patient’s baseline rather than on a population basis. Future validations and the use of integrated smartphone gait analysis may support individualized rehabilitation strategies following an ankle fracture.

## Potential Competing Interests

The authors report no competing interests.

## Ethics Statement

The study followed the Declaration of Helsinki (2024) and was approved by the Swedish Ethical Review Authority (2024-00068-01).
